# In Vivo Visualization and Quantification of Dermal Nevus Microvasculature Using Super-Resolution Ultrasound of Erythrocytes

**DOI:** 10.3390/diagnostics16152433

**Published:** 2026-08-01

**Authors:** Rikke Baarts, Ali Salari, Alexander Cuculiza Henriksen, Nathalie Sarup Panduro, Emma Kanchana Ertner Bengtsson, Niels Kvorning Ternov, Caroline Clausen, Lisbet Rosenkrantz Hölmich, Lars Lönn, Charlotte Mehlin Sørensen, Jørgen Arendt Jensen, Michael Bachmann Nielsen

**Affiliations:** 1 Department of Clinical Medicine, University of Copenhagen, 2200 Copenhagen, Denmark; 2 Department of Diagnostic Radiology, Rigshospitalet, 2100 Copenhagen, Denmark; 3 Center for Fast Ultrasound Imaging (CFU), Health Tech, Technical University of Denmark, 2800 Lyngby, Denmark; 4 Department of Plastic Surgery, Copenhagen University Hospital, Herlev and Gentofte Hospital, 2730 Herlev, Denmark; 5 Melatech, Trekronergade 126B, 2500 Copenhagen, Denmark; 6 Department of Biomedical Sciences, Division of Renal and Vascular Physiology, University of Copenhagen, 2200 Copenhagen, Denmark

**Keywords:** super-resolution ultrasound, erythrocytes, dermal microvasculature, melanocytic nevi, microvascular imaging, contrast-free ultrasound, ultrasound

## Abstract

**Background/Objectives**: Distinguishing melanoma from benign melanocytic nevi remains a central diagnostic challenge, and vascular features may provide additional information beyond surface morphology. Super-resolution ultrasound using the erythrocytes (SURE) is a contrast-free imaging technique that uses endogenous erythrocyte scattering signals to reconstruct microvascular architecture beyond the conventional diffraction limit. This study evaluated the feasibility of SURE for in vivo visualization and quantitative assessment of dermal microvasculature in clinically benign nevi. **Methods**: Eleven participants with 35 clinically benign dermal nevi were included. All lesions underwent clinical and dermoscopic assessment, conventional B-mode ultrasound, color and power Doppler imaging, and SURE imaging. Eight larger nevi were imaged in two imaging planes, resulting in 43 SURE acquisitions. SURE reconstructions were assessed qualitatively for microvascular morphology and quantitatively for vessel diameter and erythrocyte flow velocity in proximal, intermediate, and distal visible intralesional vessel segments. **Results**: Dermoscopic and conventional ultrasound images were acquired for all lesions. SURE reconstructions of sufficient quality for quantitative analysis were obtained for all included acquisitions, yielding 129 vessel diameter measurements and 129 corresponding velocity measurements. Conventional Doppler demonstrated absent or minimal detectable vascular signal in the majority of lesions, whereas SURE visualized branching structures consistent with dermal microvascular networks in all the lesions. The mean vessel diameter was 85.0 ± 20.2 µm, with measured diameters ranging from 48.1 to 173.0 µm. The median flow velocity was 1.80 [1.30–2.50] mm/s. No significant differences in vessel diameter or velocity were observed between proximal, intermediate, and distal intralesional segments. **Conclusions**: SURE enabled contrast-free in vivo visualization and quantitative assessment of low-velocity dermal microvasculature in clinically benign nevi. These findings support the feasibility of SURE for microvascular mapping of melanocytic lesions and provide a basis for future studies including malignant lesions, volumetric imaging, and histopathological validation.

## 1. Introduction

Cutaneous melanoma represents the most lethal form of skin cancer, with approximately 325,000 new cases and 57,000 deaths recorded globally in 2020 [1,2,3,4,5,6]. Incidence rates are the highest in Australia and New Zealand, followed by Northern and Western Europe, and projections estimate that annual global incidence may exceed 510,000 cases by 2040 [1,2,3,7,8]. In Denmark, age-standardized melanoma incidence has increased substantially in recent decades, with current rates remaining high in both sexes and particularly among women [9]. Incidence continues to rise, particularly among older age groups, while mortality has stabilized in younger populations in several high-income countries. This epidemiological shift is likely driven by a combination of increased cumulative ultraviolet exposure, improved diagnostic activity, and demographic aging [1,4,5,6,10,11].

Prognosis is strongly stage-dependent, underscoring the importance of early and accurate lesion characterization [2,12,13,14]. The primary diagnostic challenge lies in distinguishing melanoma from benign melanocytic nevi. Current evaluation relies predominantly on dermoscopy followed by histopathological confirmation after excision [1,2,12,13,15,16,17,18,19]. However, diagnosis is highly operator-dependent and largely limited to superficial assessment, driving a clinical tendency to biopsy or excise many lesions that turn out to be benign [16,19,20,21,22].

**Figure 1:**
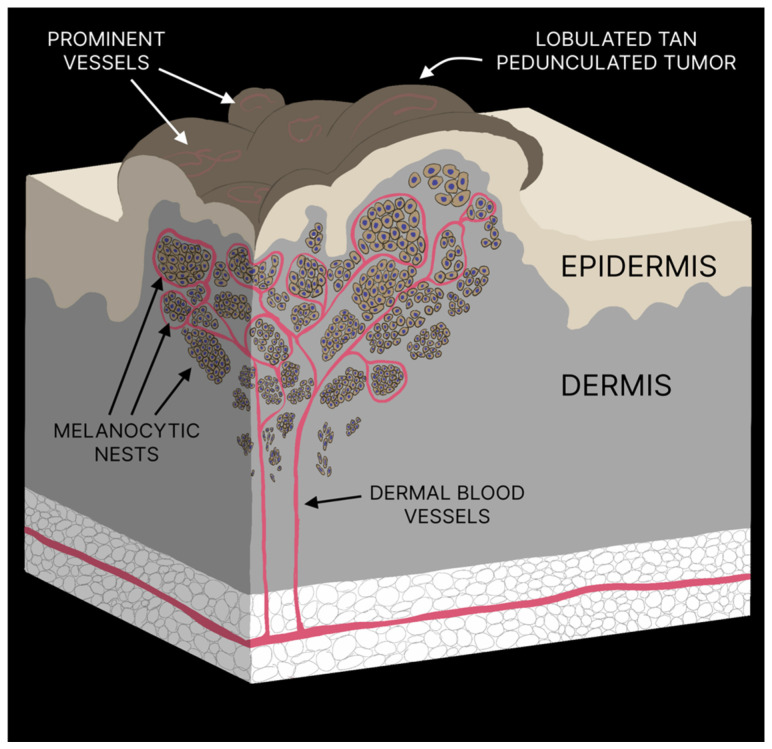
Schematic, caricatured depiction of the histological architecture of a dermal nevus. On the surface, a lobulated, tan, pedunculated tumor with prominent vessels is seen, while the dermis contains melanocytic nests arranged around the dermal blood vessels. Morphological illustration borrowed with permission from DermLoop’s Learning Library.

Microvascular architecture is increasingly recognized as a biologically relevant feature in the evaluation of melanocytic lesion, with alterations in vessel density, morphology, and flow characteristics associated with increasing Breslow thickness, ulceration, and markers of aggressive tumor biology (see Figure 1 for a detailed view on the microvasculature of a melanocytic dermal nevus) [23,24,25,26,27]. Non-invasive visualization of the dermal microvasculature remains challenging [25,28]. Conventional Doppler techniques are limited by diffraction and exhibit reduced sensitivity to low-velocity flow in small-diameter dermal vessels. Advanced microvascular imaging approaches such as photoacoustic methods and Superb Microvascular Imaging partially overcome these constraints [28,29,30].

Super-resolution ultrasound using the erythrocytes (SURE) is a novel contrast-free imaging approach that localizes and tracks individual erythrocyte scattering signals across thousands of ultrafast frames, enabling the reconstruction of microvascular architecture beyond the conventional diffraction limit [31,32,33]. A recent proof-of-concept study demonstrated the feasibility of SURE in a single melanocytic nevus [34].

We hypothesized that SURE would enable contrast-free visualization of dermal nevus microvasculature and allow the quantification of small-vessel diameter and low-velocity erythrocyte flow in benign dermal nevi, including microvascular structures that remain poorly detectable using conventional Doppler ultrasound.

## 2. Materials and Methods

This study included vessel diameter measurements and velocity measurements obtained from the first 35 dermal nevi of 11 participants (10 with melanocytic and 1 with vascular nevi). The study was approved by the Regional Committee on Health Research Ethics for the Capital Region of Denmark (VEK number: H-24062143) and was conducted in accordance with the Declaration of Helsinki. Written informed consent was obtained prior to participation. Eligible participants were healthy adults aged 18 years or older with clinically stable dermal cutaneous lesions that patients reported had remained morphologically unchanged for at least three years. Exclusion criteria included a prior diagnosis or current suspicion of malignant melanoma, symptomatic lesions (e.g., pain, pruritus, or irritation), recent morphological changes, pregnancy, or anatomical inaccessibility of the lesion for scanning. Recruitment was conducted through public advertisements.

### 2.1. Study Design

This study was designed as a technical feasibility study evaluating whether contrast-free super-resolution ultrasound using erythrocytes (SURE) can be applied in vivo for the visualization and quantitative assessment of microvascular structures in benign dermal nevi. In accordance with established principles for feasibility study design, the study focused on practical implementation, image acquisition feasibility, and limited quantitative assessment rather than diagnostic accuracy or clinical decision-making [35]. Specifically, we assessed whether SURE imaging could be performed in a clinical setting, whether reconstructed microvascular images of sufficient quality could be obtained, and whether vessel diameter and erythrocyte flow velocity could be extracted from selected intralesional vessels.

### 2.2. Examination Process

Prior to scanning, participants received written and oral information reiterating the study procedures. Baseline demographic and anthropometric data were recorded, including age, weight, and height (Table 1). Skin phototype was assessed using the Fitzpatrick scale, and participants were interviewed regarding sun exposure habits, including history of sunburn and solarium use. A clinical examination was subsequently performed by a physician to identify eligible melanocytic nevi. All identified lesions were photographed using the platform Dermloop (Melatech ApS, Valby, Denmark), including a clinical and a dermoscopic photo, a location marked on a 3D avatar and a short medical history. Lesions were reviewed by two experts with more than six years of experience in evaluating melanocytic and non-melanocytic skin lesions. Only benign nevi without any malignancy suspicious criteria were included in the study.

### 2.3. Scanning Procedure

**Figure 2:**
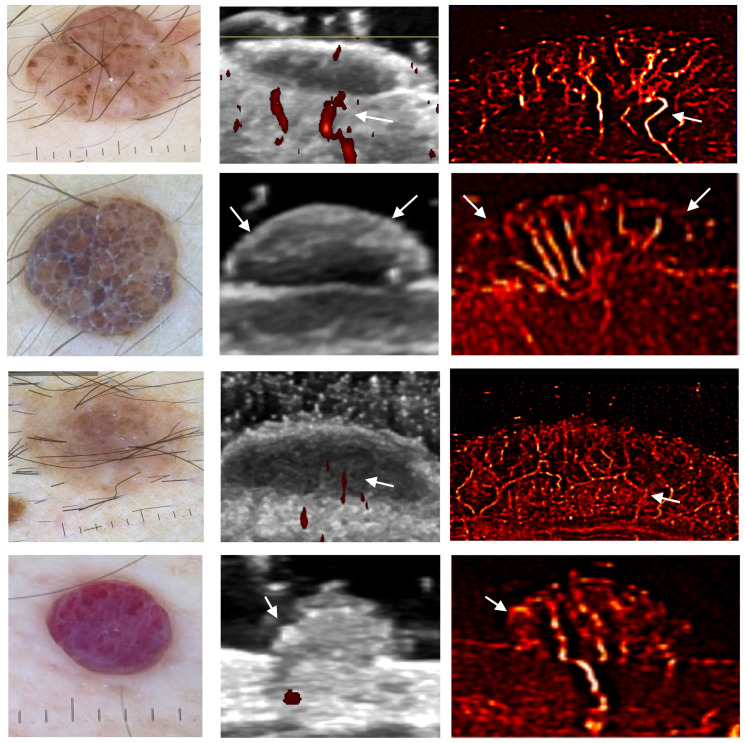
The figure shows four representative benign dermal nevi arranged in rows (the first 3 are melanocytic and the last vascular). The first column displays dermoscopic images used for clinical lesion assessment and diagnostic characterization, with a millimeter scale included for size reference. The second column shows corresponding power Doppler ultrasound images, demonstrating absent or only sparse detectable vascular signal within the lesions. The third column shows the corresponding super-resolution ultrasound using the erythrocytes (SURE) reconstructions, revealing detailed microvascular architecture with visualization of small-caliber dermal vessels extending throughout the lesions. White arrows indicate approximate corresponding regions across the ultrasound images to facilitate visual comparison between conventional Doppler and SURE.

The scanning region was selected based on clinical inspection, and the images and location were documented within the Dermloop platform. Each lesion was subsequently imaged using two ultrasound platforms for comparative evaluation. A conventional clinical ultrasound system, GE LOGIQ E9 (GE HealthCare, Chicago, IL, USA), was used to perform standard vascular assessments with color and power Doppler imaging (Figure 2). When obtaining Doppler images, the setting was aligned for the lowest scale at which a representative flow was presented without too much interference.

For SURE imaging, a Verasonics Vantage 256 research platform (Verasonics, Inc., Kirkland, WA, USA) was employed. The same linear transducer, GE L8-18i-D(GE HealthCare, Chicago, IL, USA), was used for both acquisitions. A custom-designed probe holder was developed to stabilize the transducer, minimize operator-induced motion and image the same anatomical imaging plane, enabling a direct comparison between acquisition modalities. For the SURE protocol, synthetic aperture ultrasound sequences were implemented, achieving a frame rate of 208 Hz. The SURE sequence used a nominal transmit frequency of 10 MHz; the measured center frequency of the transmitted pulse was 9.29 MHz, corresponding to a wavelength of approximately 166 µm in tissue (assuming a sound speed of 1540 m/s).

Before the study, comprehensive safety evaluations were conducted, including thermal, acoustic intensity, and electrical assessments to ensure compliance with safety standards. The measured mechanical index (MI) was below 0.5 (FDA limit: 1.9), and the derated spatial-peak temporal-average intensity (I_spta) was below 7.5 mW/cm^2^ (FDA limit: 720 mW/cm^2^).

### 2.4. SURE Imaging Reconstruction

After acquisition, SURE scan data were processed in Matlab 2021b (MathWorks, Natick, MA, USA) using a locally developed post-processing program at the Center for Fast Ultrasound Imaging (CFU), Technical University of Denmark. For each lesion, 10 s of ultrasound data was acquired and processed through the established local SURE pipeline. If motion artifacts were present, affected frames or segments were excluded during post-processing, and reconstruction was performed using the remaining data with sufficient image stability. Minor scan-specific adjustments were performed when required, including corrections related to lesion position and clutter suppression. Each reconstructed SURE image was visually assessed to ensure adequate quality. Beamforming was performed using a GPU-accelerated implementation in MATLAB R2021b. Transverse oscillation-based motion estimation was applied to estimate and compensate for tissue motion, and singular value decomposition (SVD) filtering was used to suppress stationary tissue clutter while preserving blood flow components. The amplitude and spatial location of signal peaks in the filtered data were used to generate SURE intensity images representing the spatial distribution of erythrocytes, and a recursive nearest-neighbor tracking algorithm was applied to estimate flow velocity and generate the corresponding velocity maps. For more in depth explanation on the SURE pipeline please see the following references [31,33,34,36]. Previous validation of the SURE method demonstrated a spatial resolution of approximately 29 µm [33], supporting its ability to visualize vascular structures substantially smaller than the acoustic wavelength.

**Figure 3:**
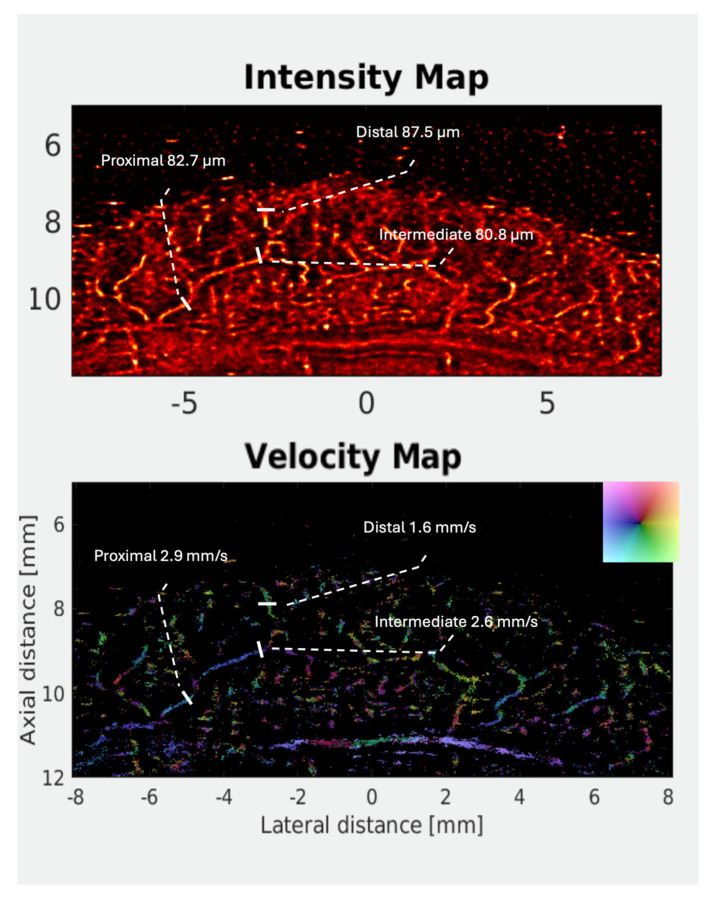
Representative quantitative SURE analysis of dermal microvasculature. The reconstructed branching microvascular network allows localized assessment of vessel diameter and erythrocyte flow velocity. The upper panel shows the intensity map, with white line segments indicating the sites of vessel diameter measurements. The corresponding vessel diameters are displayed directly on the image in micrometers (µm). The lower panel shows the velocity map, where white line segments indicate the sites of velocity measurements, with corresponding flow velocities displayed in millimeters per second (mm/s). In both panels, the x-axis represents lateral distance in millimeters (mm), and the y-axis represents axial distance in millimeters (mm).

From the processed SURE data, secondary reconstructions were generated as intensity and velocity maps. For quantitative analysis, the SURE imaging plane and time segment with the least motion and highest reconstruction quality were selected. Vessel measurements were performed manually by a trained physician on vessels located within the dermal nevus. Measurement points were categorized according to visible intralesional vessel segment as proximal, intermediate, or distal vessels, as illustrated in Figure 3. Proximal segments were defined as the first third of the visible vessel or the segment before the first branch point, when identifiable. Distal segments were defined as the most peripheral visible vessel segment or the segment beyond the last identifiable branch point. Intermediate segments comprised the remaining vessel course between proximal and distal segments. This classification referred to the visible course of individual vessel segments within the two-dimensional SURE reconstruction and did not represent depth from the skin surface or distance from the center of the nevus.

Vessel diameter was estimated directly from the SURE intensity maps. For each selected vessel, an intensity profile was obtained perpendicular to the vessel axis at the place of highest visual quality, ensuring optimal data quality. Vessel diameter was defined as the full width at half maximum (FWHM) of the normalized intensity profile. Blood flow velocity was obtained from the corresponding SURE velocity maps by selecting the same vessel segment, and the peak velocity was recorded for each measurement point.

### 2.5. Statistical Analysis

Statistical analyses were performed using R2025.09.2+418 (R Foundation for Statistical Computing, Vienna, Austria). Distributional assumptions were assessed by visual inspection of histograms and Q-Q plots. Microvascular measurements were categorized according to intralesional vessel segment as proximal, intermediate, and distal. Vessel diameter was summarized as mean ± standard deviation, while blood flow velocity was summarized as median with interquartile range due to right-skewed distribution.

Vessel diameter and blood flow velocity were analyzed using separate mixed-effects models with intralesional vessel segment as fixed effect and nevus identity as random intercept to account for repeated measurements within the same lesion. Blood flow velocity was log-transformed prior to model fitting to improve residual normality. Overall differences between locations were assessed from the mixed-effects models, and proximal location was used as the reference for location-specific comparisons. Descriptive values are presented as observed mean ± standard deviation for vessel diameter and observed median [IQ range] for blood flow velocity. Model-based *p*-values are reported for overall and location-specific comparisons. A *p*-value below 0.05 was considered statistically significant.

## 3. Results

A total of 11 participants with 35 dermal nevi were included (Table 1). In total, 27 nevi contributed to one SURE acquisition each and 8 larger nevi contributed two acquisitions each, yielding a total of 43 SURE acquisitions. No nevi were suspected of malignancy based on medical history and dermoscopy. Conventional ultrasound (B-mode, color Doppler, and power Doppler) and dermoscopic images were successfully acquired for all lesions. SURE reconstructions of sufficient quality were obtained from all included acquisitions, resulting in 129 vessel diameter measurements and 129 corresponding velocity measurements across proximal, intermediate and distal intralesional segments. Conventional Doppler demonstrated absent or minimal detectable flow in the majority of lesions.

SURE imaging repeatedly demonstrated branching structures consistent with microvascular network, with clearly delineated vessel trajectories extending throughout the dermal nevi. Vessels below 100 μm in diameter were repeatedly resolved, with an overall mean vessel diameter of 85.0 ± 20.2 μm, while the overall median flow velocity was 1.80 [1.30–2.50] mm/s (Table 2).

To assess whether measurements varied depending on where along the visible vessel course they were sampled, diameter and velocity were recorded at three points per lesion (proximal, intermediate, distal; see Methods). Measurements were closely similar across the three sampling points (diameters: 89.4 ± 23.4, 82.7 ± 19.0, 83.0 ± 17.4 µm; *p* = 0.165; velocities: 1.90 [1.20–2.95], 1.90 [1.45–2.50], 1.70 [1.30–2.30] mm/s; *p* = 0.591), indicating that measurements were consistent regardless of sampling location within the lesion (Table 2).

## 4. Discussion

A recent proof-of-concept study demonstrated the feasibility of SURE in a single melanocytic nevus [34]. In recent years, SURE has been applied to other human tissues, including lymph nodes and tendons, with similar findings; however, most of these studies are based on single participant cases [37,38,39,40]. The present study extends this work by presenting the first systematic in-human application of super-resolution ultrasound using the erythrocytes (SURE) for the visualization and quantitative assessment of dermal nevus microvasculature in 35 nevi from 11 participants. SURE resolved structures consistent with microvascular architecture below 100 μm and detected flow velocities in the low millimeter-per-second range across benign dermal nevi. Conventional color and power Doppler demonstrated absent or minimal vascular signal in most lesions, consistent with the known limitations of flow-sensitive ultrasound techniques in dermal microvascular imaging. Beyond simple vessel detection, SURE enabled the visualization of organized intralesional microvascular networks with a similar branching morphology observed across lesions. These findings demonstrate the feasibility of SURE for in vivo characterization of dermal microcirculation.

The branching networks visualized with SURE are biologically plausible in benign dermal nevi. The vessel dimensions and flow velocities observed in the present study are consistent with the expected scale of superficial dermal microcirculation, in which vessels are reported to range approximately from 50 to 150 µm in dermis and below 50 µm in the superficial plexus, with vertically oriented branching vessels connecting the superficial and deep horizontal plexuses (see Figure 1) [29]. For comparison, high-frequency ultrafast ultrasound studies on human skin have previously reported detectable dermal microvascular diameters in a similar range, including microvessels of approximately 141–153 µm in healthy dorsal hand skin and detectable diameters from 40.69 µm to more than 1 mm in keloid tissue (the upper range reflecting larger feeding vessels within the highly vascularized scar tissue rather than true microvasculature) [29,41,42,43]. This study found a mean vessel diameter of 85.0 ± 20.2 µm and the smallest measured vessels of 48.1 µm. The measured SURE data therefore fall within a biologically plausible range for dermal microvascular structures as well as previously measured microvasculature structures (Table 3).

The measured flow velocities were likewise within the low millimeter-per-second range expected for superficial cutaneous microcirculation. Dermal vessels are known to be challenging for conventional Doppler imaging because of their small caliber, superficial location, and slow flow. For comparison, previously reported values include very low velocities of approximately 0.5 mm/s in 100–300 µm vessels in cutaneous microcirculation, as well as velocities of 1–4 mm/s in nailfold microvasculature [43,45]. The median SURE-derived velocity of 1.80 [1.30–2.50] mm/s is therefore physiologically plausible and supports the interpretation that the reconstructed vascular signals represent slow dermal microvascular flow rather than macroscopic vascular structures.

Conventional Doppler ultrasound is challenged in the assessment of dermal microcirculation due to the combination of extremely small vessel caliber and very low flow velocities [29,30,46,47,48]. Conventional Doppler and microvascular Doppler techniques depend on clutter suppression to separate blood flow from tissue signal. However, tissue clutter is a high-energy, predominantly low-velocity signal, and slow microvascular blood flow may overlap with this signal in the low-velocity spectral range. Aggressive clutter filtering may therefore suppress slow blood flow together with tissue motion, reducing sensitivity to physiological dermal microvascular flow [49,50,51]. In the present study, this limitation was reflected by absent or minimal vascular signal on color and power Doppler despite clearly visible microvascular networks on SURE (Figure 2).

Recent advances in microvascular ultrasound imaging, such as Superb Microvascular Imaging (SMI), have partly addressed these challenges through improved clutter filtering and increased sensitivity to slow flow within small-caliber vessels [29,49,52]. Several studies have demonstrated improved the visualization of dermal vascularization using these approaches compared with conventional color and power Doppler imaging [29,30,46,50,53]. Nevertheless, these techniques remain Doppler-based and rely on flow-sensitive signal detection rather than localization-based reconstruction of individual intravascular scatterers [31,32,33,54,55]. In contrast, super-resolution ultrasound techniques achieve sub-diffraction imaging by repeatedly localizing and tracking isolated scatterers across ultrafast acquisitions [32,54,56,57]. Unlike previously described super-resolution ultrasound approaches that rely on intravenously administered microbubble contrast agents, SURE utilizes endogenous erythrocyte scattering signals as native intravascular tracers [31,33,34,40].

Vessel diameter and velocity were sampled at three points in each nevus as a check of measurement consistency. The measurements showed no significant positional differences across proximal, intermediate and distal regions. The absence of significant differences indicates that measurements did not depend on where along the vessel course they were obtained. This should not be interpreted as evidence for or against a true proximal-to-distal anatomical gradient, since the classification reflected the visible two-dimensional vessel course rather than histologically verified parent–daughter branching relationships. The lack of a proximal-to-distal gradient may therefore reflect both the relatively organized vascular pattern of benign nevi and methodological factors such as two-dimensional sampling, vessel tortuosity, mixed vessel types, and manual selection of measurable segments.

Several limitations should be acknowledged. First, this was a preliminary feasibility study with a relatively small cohort consisting exclusively of benign dermal nevi, and no histopathological correlation was performed. Consequently, the present findings do not allow conclusions regarding diagnostic performance or discrimination between benign and malignant lesions. The underlying SURE technique has previously been validated against independent anatomical and technique-based references, including ex vivo micro-computed tomography and concordant flow profiles with microbubble-based ultrasound localization microscopy in animal models [33,36,58]. However, the present study did not include an independent anatomical reference standard (e.g., histopathology or co-registered optical coherence tomography angiography) specific to human dermal nevi, and direct validation of the technique in this tissue and clinical context remains an important objective for future work. In the present study, SURE imaging was analyzed in selected two-dimensional imaging planes and therefore did not provide volumetric assessment of the entire nevus. This should be regarded as a limitation of the current study design rather than an inherent limitation of the SURE technique. Third, quantitative vascular analysis was based on a limited number of manually selected vessel segments, which may introduce observer dependency and sampling bias. Vessel selection and measurement were performed by a single trained observer, and intra- or inter-observer reliability was not assessed. As vessel segments were selected at the location of highest visual image quality, this introduces a potential selection bias, and the reported measurements should be interpreted as illustrative of the achievable range rather than a systematically sampled or fully reproducible estimate. Future studies should include formal reliability assessment (e.g., repeated measurements by independent observers). In addition, super-resolution ultrasound remains technically demanding and currently requires high-frame-rate acquisitions combined with computationally intensive offline processing, which limits real-time clinical implementation. Finally, comparison was limited to conventional color and power Doppler, and future studies should include comparison with advanced microvascular Doppler techniques such as Superb Microvascular Imaging. Larger studies including malignant lesions, histopathological validation, standardized acquisition protocols, and automated quantitative analysis will therefore be important for future translation into clinical practice. Importantly, future clinical studies evaluating diagnostic accuracy should be aligned with the STARD guidelines to support translation into clinical practice [59].

## 5. Conclusions

In conclusion, SURE enabled contrast-free visualization and quantitative assessment of dermal microvasculature in benign nevi. The technique resolved structures consistent with low-velocity microvascular architecture that remained largely undetectable using conventional Doppler imaging and demonstrated similar branching patterns across dermal nevi. These findings support the foundation for future studies investigating whether microvascular architecture may contribute to non-invasive differentiation and biological characterization of melanocytic skin lesions.

## Figures and Tables

**Table 1:** Demographics and characteristics.

**Participant Demographics**	
**Characteristics**	**Value**
Participants, *n*	11
Age, years	54.5 ± 12.2 (26–69)
Females	7 (63.6%)
Males	4 (36.4%)
Fitzpatrick skin type I	2 (18.2%)
Fitzpatrick skin type II	8 (72.7%)
Fitzpatrick skin type III	1 (9.1%)
**Lesion and Measurement Characteristics**	
**Characteristics**	**Value**
Nevi	35
Lesion diameter, mm	7.7 ± 3.4 (3.0–16.0)
SURE scans, *n*	43
Vessel diameter measurements, *n*	129
Velocity measurements, *n*	129
Vessel diameter range, µm	48.1–173.0
Velocity range, mm/s	0.6–6.3

Age is presented as mean ± standard deviation (range). Vessel diameter and velocity measurements refer to intralesional measurements obtained across proximal, intermediate and distal vessel segments. Lesion diameter is presented as mean ± standard deviation (range). Vessel diameter and velocity ranges are presented as minimum–maximum.

**Table 2:** Microvascular measurements by intralesional location.

Variable	Statistics	Overall	Proximal	Intermediate	Distal
Velocity, mm/s	Median [IQR]	1.80 [1.30–2.50]	1.90 [1.20–2.95]	1.90 [1.45–2.50]	1.70 [1.30–2.30]
		*n* = 129	*n* = 43	*n* = 43	*n* = 43
		*p* = 0.591	Reference	*p* = 0.985	*p* = 0.707
Vessel diameter, µm	Mean ± SD	85.0 ± 20.2	89.4 ± 23.4	82.7 ± 19.0	83.0 ± 17.4
		*n* = 129	*n* = 43	*n* = 43	*n* = 43
		*p* = 0.165	Reference	*p* = 0.209	*p* = 0.247

Velocity is presented as median [IQR] and was analyzed using a log-transformed mixed-effects model. Vessel diameter is presented as mean ± SD and was analyzed using a linear mixed-effects model. Nevus identity was included as a random intercept. Overall *p*-values represent the fixed effect of location. Pairwise *p*-values are Tukey-adjusted comparisons with proximal as reference.

**Table 3:** Comparison of SURE and related contrast-free super-resolution ultrasound microvascular imaging studies.

Study	Technique	Tissue	Subjects	Smallest Vessel Resolved	Velocity Range
Present study	SURE	Human, dermal nevi	11 participants (35 nevi)	48.1 µm mean 85.0 ± 20.2 µm	1.80 [1.30–2.50] mm/s (median)
Salari et al. 2025 [34] *	SURE	Human, melanocytic mole	1 participant	65 µm	As low as 3.2 mm/s
Naji et al. 2025 [40] *	SURE	Human, lymph node	4 lymph nodes	40 µm	2–15 mm/s
Naji et al. 2024 [39] *	SURE	Human, axillary lymph node	1 lymph node	67 µm	Up to 16.4 mm/s
Tabatabaei Majd et al. 2025 [37] *	SURE	Human, injured patellar tendon	-	44 µm	-
Naji et al. 2025 [38] *	SURE	Rat, liver and kidney	1 rat	53.3 µm	4–10 mm/s
Li et al. 2026 [44] *	Contrast-free eULM	Cerebrovasculature	-	7.3 µm	-

* Vessel diameters and velocities are reported as described in the cited studies; direct comparison across tissues, species, and acquisition protocols should be made with caution.

## Data Availability

The datasets presented in this article are not readily available because they are stored at DTU and contain data subject to ethical and privacy restrictions. The data are therefore not publicly available. Requests to access the datasets should be directed to Michael Bachmann Nielsen [michael.bachmann.nielsen@regionh.dk] or to Rikke Baarts [rjoh0026@regionh.dk], and will be considered on a case-by-case basis in accordance with the ethical approval, participant consent, applicable data protection regulations, DTU policies, and policies of the hospital.

## Abbreviations

The following abbreviations are used in this manuscript:

CFU	Center for Fast Ultrasound Imaging
ERC	European Research Council
IQR	Interquartile range
SMI	Superb microvascular imaging
STARD	Standards for the Reporting of Diagnostic Accuracy Studies
SURE	Super-resolution ultrasound using the erythrocytes

## References

[1] Helgadottir H., Mikiver R., Schultz K., Nielsen K., Portelli F., Lapins J., Puig S., Isaksson K. (2024). Melanoma Incidence and Mortality Trends Among Patients Aged 59 Years or Younger in Sweden. JAMA Dermatol..

[2] Garbe C., Amaral T., Peris K., Hauschild A., Arenberger P., Basset-Seguin N., Bastholt L., Bataille V., Brochez L., Del Marmol V. (2025). European Consensus-Based Interdisciplinary Guideline for Melanoma. Part 1: Diagnostics—Update 2024. Eur. J. Cancer.

[3] Arnold M., Singh D., Laversanne M., Vignat J., Vaccarella S., Meheus F., Cust A.E., De Vries E., Whiteman D.C., Bray F. (2022). Global Burden of Cutaneous Melanoma in 2020 and Projections to 2040. JAMA Dermatol..

[4] Huang J., Chan S.C., Ko S., Lok V., Zhang L., Lin X., Lucero-Prisno D.E., Xu W., Zheng Z.-J., Elcarte E. (2023). Global Incidence, Mortality, Risk Factors and Trends of Melanoma: A Systematic Analysis of Registries. Am. J. Clin. Dermatol..

[5] Sun Y., Shen Y., Liu Q., Zhang H., Jia L., Chai Y., Jiang H., Wu M., Li Y. (2025). Global Trends in Melanoma Burden: A Comprehensive Analysis from the Global Burden of Disease Study, 1990–2021. J. Am. Acad. Dermatol..

[6] Liu C., Liu X., Hu L., Li X., Xin H., Zhu S. (2024). Global, Regional, and National Burden of Cutaneous Malignant Melanoma from 1990 to 2021 and Prediction to 2045. Front. Oncol..

[7] Naik P.P. (2021). Cutaneous Malignant Melanoma: A Review of Early Diagnosis and Management. World J. Oncol..

[8] Hwang J.C., Peacker B.L., Hartman R.I. (2025). Screening and Novel Diagnostic Technologies for Melanoma: An Update. Melanoma Manag..

[9] International Agency for Research on Cancer (2026). Global Cancer Observatory: Cancer Over Time. Melanoma of Skin, Denmark.

[10] Wang X.M., Borsky K., Proctor D.W., Goodall R., Marshall D.C., Dobell W., Salciccioli J.D., Matin R.N., Shalhoub J., El-Muttardi N. (2025). Trends in Cutaneous Melanoma Mortality and Incidence in European Union 15+ Countries between 1990 and 2019. Acad. Dermatol. Venereol..

[11] Sendín-Martín M., Bueno-Molina R.C., Hernández-Rodríguez J.-C., Cayuela L., Cayuela A., Pereyra-Rodríguez J.-J. (2025). Trends in Cutaneous Malignant Melanoma Mortality in Europe from 1992 to 2021. Clin. Exp. Dermatol..

[12] Garbe C., Amaral T., Peris K., Hauschild A., Arenberger P., Basset-Seguin N., Bastholt L., Bataille V., Brochez L., Del Marmol V. (2025). European Consensus-Based Interdisciplinary Guideline for Melanoma. Part 2: Treatment—Update 2024. Eur. J. Cancer.

[13] Falk Delgado A., Zommorodi S., Falk Delgado A. (2019). Sentinel Lymph Node Biopsy and Complete Lymph Node Dissection for Melanoma. Curr. Oncol. Rep..

[14] Wong S.L., Faries M.B., Kennedy E.B., Agarwala S.S., Akhurst T.J., Ariyan C., Balch C.M., Berman B.S., Cochran A., Delman K.A. (2018). Sentinel Lymph Node Biopsy and Management of Regional Lymph Nodes in Melanoma: American Society of Clinical Oncology and Society of Surgical Oncology Clinical Practice Guideline Update. Ann. Surg. Oncol..

[15] Gershenwald J.E., Scolyer R.A. (2018). Melanoma Staging: American Joint Committee on Cancer (AJCC) 8th Edition and Beyond. Ann. Surg. Oncol..

[16] Dinnes J., Deeks J.J., Chuchu N., Ferrante Di Ruffano L., Matin R.N., Thomson D.R., Wong K.Y., Aldridge R.B., Abbott R., Fawzy M. (2018). Dermoscopy, with and without Visual Inspection, for Diagnosing Melanoma in Adults. Cochrane Database Syst. Rev..

[17] Vestergaard M.E., Macaskill P., Holt P.E., Menzies S.W. (2008). Dermoscopy Compared with Naked Eye Examination for the Diagnosis of Primary Melanoma: A Meta-Analysis of Studies Performed in a Clinical Setting. Br. J. Dermatol..

[18] Bafounta M.-L., Beauchet A., Aegerter P., Saiag P. (2001). Is Dermoscopy (Epiluminescence Microscopy) Useful for the Diagnosis of Melanoma?: Results of a Meta-Analysis Using Techniques Adapted to the Evaluation of Diagnostic Tests. Arch. Dermatol..

[19] Wolner Z.J., Yélamos O., Liopyris K., Rogers T., Marchetti M.A., Marghoob A.A. (2017). Enhancing Skin Cancer Diagnosis with Dermoscopy. Dermatol. Clin..

[20] Petty A.J., Ackerson B., Garza R., Peterson M., Liu B., Green C., Pavlis M. (2020). Meta-Analysis of Number Needed to Treat for Diagnosis of Melanoma by Clinical Setting. J. Am. Acad. Dermatol..

[21] Nelson K.C., Swetter S.M., Saboda K., Chen S.C., Curiel-Lewandrowski C. (2019). Evaluation of the Number-Needed-to-Biopsy Metric for the Diagnosis of Cutaneous Melanoma: A Systematic Review and Meta-Analysis. JAMA Dermatol..

[22] Chen J.Y., Fernandez K., Fadadu R.P., Reddy R., Kim M.-O., Tan J., Wei M.L. (2025). Skin Cancer Diagnosis by Lesion, Physician, and Examination Type: A Systematic Review and Meta-Analysis. JAMA Dermatol..

[23] Sharma S., Sharma M.C., Sarkar C. (2005). Morphology of Angiogenesis in Human Cancer: A Conceptual Overview, Histoprognostic Perspective and Significance of Neoangiogenesis. Histopathology.

[24] Schuh S., Sattler E.C., Rubeck A., Schiele S., De Carvalho N., Themstrup L., Ulrich M., Jemec G.B.E., Holmes J., Pellacani G. (2023). Dynamic Optical Coherence Tomography of Blood Vessels in Cutaneous Melanoma-Correlation with Histology, Immunohistochemistry and Dermoscopy. Cancers.

[25] Podolec K., Bronikowska A., Pirowska M., Wojas-Pelc A. (2020). Dermoscopic Features in Different Dermatopathological Stages of Cutaneous Melanomas. Postep. Dermatol. Alergol..

[26] Perivoliotis K., Ntellas P., Dadouli K., Koutoukoglou P., Ioannou M., Tepetes K. (2017). Microvessel Density in Patients with Cutaneous Melanoma: An Up-to-Date Systematic Review and Meta-Analysis. J. Ski. Cancer.

[27] De Pinto G., Mignozzi S., La Vecchia C., Levi F., Negri E., Santucci C. (2024). Global Trends in Cutaneous Malignant Melanoma Incidence and Mortality. Melanoma Res..

[28] Baarts R., Henriksen A.C., Panduro N.S., Bengtsson E.K.E., Salari A., Clausen C., Hölmich L.R., Lönn L., Sørensen C.M., Jensen J.A. (2026). Ultrasound-Based Techniques for Visualization of Dermal Microvasculature: A Scoping Review. Diagnostics.

[29] Corvino A., Varelli C., Cocco G., Corvino F., Catalano O. (2022). Seeing the Unseen with Superb Microvascular Imaging: Ultrasound Depiction of Normal Dermis Vessels. J. Clin. Ultrasound.

[30] Catalano O., Corvino A., Basile L., Catalano F., Varelli C. (2023). Use of New Microcirculation Software Allows the Demonstration of Dermis Vascularization. J. Ultrasound.

[31] Jensen J.A., Schou M., Andersen S.B., Søgaard S.B., Sørensen C.M., Nielsen M.B., Gundlach C., Kjer H.M., Dahl A.B., Stuart M.B., Ruiter N.V., Bottenus N. (2022). Fast Super Resolution Ultrasound Imaging Using the Erythrocytes. Proceedings of the Medical Imaging 2022: Ultrasonic Imaging and Tomography.

[32] Christensen-Jeffries K., Couture O., Dayton P.A., Eldar Y.C., Hynynen K., Kiessling F., O’Reilly M., Pinton G.F., Schmitz G., Tang M.-X. (2020). Super-Resolution Ultrasound Imaging. Ultrasound Med. Biol..

[33] Arendt Jensen J., Amin Naji M., Kazmarek Præsius S., Taghavi I., Schou M., Naur Hansen L., Bech Andersen S., Byrholdt Søgaard S., Sarup Panduro N., Mehlin Sørensen C. (2024). Super-Resolution Ultrasound Imaging Using the Erythrocytes—Part I: Density Images. IEEE Trans. Ultrason. Ferroelectr. Freq. Control.

[34] Salari A., Baarts R., Naji M.A., Tomov B.G., Nielsen M.B., Jensen J.A. (2025). Human Mole Contrast-Free Microvascular Imaging Using Erythrocytes. 2025 IEEE International Ultrasonics Symposium (IUS).

[35] Bowen D.J., Kreuter M., Spring B., Cofta-Woerpel L., Linnan L., Weiner D., Bakken S., Kaplan C.P., Squiers L., Fabrizio C. (2009). How We Design Feasibility Studies. Am. J. Prev. Med..

[36] Naji M.A., Taghavi I., Schou M., Præsius S.K., Hansen L.N., Panduro N.S., Andersen S.B., Søgaard S.B., Gundlach C., Kjer H.M. (2024). Super-Resolution Ultrasound Imaging Using the Erythrocytes—Part II: Velocity Images. IEEE Trans. Ultrason. Ferroelectr. Freq. Control.

[37] Tabatabaei M., Bruggebusch Svensson R., Amin-Naji M., Salari A., Rath A., Kjær M., Jensen J.A., Mehrmohammadi M., Boehm C. (2025). Tendon Microvascular Structure Imaging Using Contrast-Free Super-Resolution Ultrasound in Humans. Proceedings of the Medical Imaging 2025: Ultrasonic Imaging and Tomography.

[38] Naji M.A., Taghavi I., McDermott A., Tomov B.G., Nielsen M.B., Sørensen C.M., Arendt Jensen J. (2025). Transcutaneous Microvascular Imaging of Rat Liver and Kidney Using Dropped-Frame Contrast-Free Super-Resolution Ultrasound. 2025 IEEE International Ultrasonics Symposium (IUS).

[39] Naji M.A., Sarup Panduro N., Tabatabaei Majd S.M.M., Salari A., Nielsen M.B., Mehlin Sørensen C., Jensen J.A. (2024). Super-Resolution Ultrasound Imaging Using Erythrocytes on an Axillary Human Lymph Node. 2024 IEEE Ultrasonics, Ferroelectrics, and Frequency Control Joint Symposium (UFFC-JS).

[40] Amin Naji M., Panduro N.S., Tabatabaei Majd S.M.M., Hansen L.N., Taghavi I., McDermott A., Gundlach C., Tomov B.G., Nielsen M.B., Sørensen C.M. (2025). Human Lymph Node Microvascular Imaging Using a Fast Contrast-Free Super-Resolution Ultrasound Technique. Sci. Rep..

[41] Chen P.Y., Hsueh Y.Y., Huang C.C. (2017). In Vivo Microcirculation Mapping of Human Skin Keloid by 40-MHz Ultrafast Ultrasound Imaging. 2017 IEEE International Ultrasonics Symposium (IUS).

[42] Bhatti A., Ishii T., Lafond M., Saijo Y. (2025). Volumetric Visualization of the Dermal Vasculature with Signal and Image-Based Feature Extraction on a High-Frequency Ultrafast Ultrasound Dataset. Ultrasound Med. Biol..

[43] Saijo Y., Akaishi S., Kuwahara H. (2024). High-Frequency Power Doppler Ultrasonography in Predicting Burn Depth: A Preliminary Case Report. Plast. Reconstr. Surg.-Glob. Open.

[44] Li C.-W., Huang H., Lin C.-Y., Chen D.-Q., Chuang Y.-H., Chen C., Huang C.-C. (2026). Contrast-Free Super-Resolution Microvascular Imaging through High Frequency Ultrasound Imaging. Ultrasonics.

[45] Immer F.F., Seiler A.M., Aeschbacher B.C., Mahler F., Saner H., Saner H. (2000). Influence of the Ultrasound Contrast Agent Levovist^®^ on Human Nailfold Capillary Microcirculation. Angiology.

[46] Catalano O., Masciotra A.P. (2025). Update on Newer Ultrasound Systems to Study the Microvasculature. Radiol. Med..

[47] Giavedoni P., Romaní J., De Cabo F., García-Martínez F.J., Quintana-Codina M., Roè-Crespo E., Fuertes De Vega I., Soria-Gili X., Aguayo-Ortiz R., Garbayo-Salmons P. (2025). Advanced Doppler Ultrasound Insights: A Multicenter Prospective Study on Healthy Skin. Diagnostics.

[48] Bruce M., Hannah A., Hammond R., Khaing Z.Z., Tremblay-Darveau C., Burns P.N., Hofstetter C.P. (2020). High-Frequency Nonlinear Doppler Contrast-Enhanced Ultrasound Imaging of Blood Flow. IEEE Trans. Ultrason. Ferroelectr. Freq. Control.

[49] Deegan A.J., Wang R.K. (2019). Microvascular Imaging of the Skin. Phys. Med. Biol..

[50] Babington E.A., Amedu C., Anyasor E., Reeve R. (2024). Non-Contrast Ultrasound Assessment of Blood Flow in Clinical Practice. J. Ultrason..

[51] Aziz M.U., Eisenbrey J.R., Deganello A., Zahid M., Sharbidre K., Sidhu P., Robbin M.L. (2022). Microvascular Flow Imaging: A State-of-the-Art Review of Clinical Use and Promise. Radiology.

[52] Seddiki R., Mirault T., Sitruk J., Mohamedi N., Messas E., Pernot M., Baranger J., Goudot G. (2025). Advancements in Noncontrast Ultrasound Imaging for Low-Velocity Flow: A Technical Review and Clinical Applications in Vascular Medicine. Ultrasound Med. Biol..

[53] Aghabaglou F., Ainechi A., Abramson H., Curry E., Kaovasia T.P., Kamal S., Acord M., Mahapatra S., Pustavoitau A., Smith B. (2022). Ultrasound Monitoring of Microcirculation: An Original Study from the Laboratory Bench to the Clinic. Microcirculation.

[54] Viessmann O.M., Eckersley R.J., Christensen-Jeffries K., Tang M.X., Dunsby C. (2013). Acoustic Super-Resolution with Ultrasound and Microbubbles. Phys. Med. Biol..

[55] Diamantis K., Greenaway A.H., Anderson T., Jensen J.A., Dalgarno P.A., Sboros V. (2018). Super-Resolution Axial Localization of Ultrasound Scatter Using Multi-Focal Imaging. IEEE Trans. Biomed. Eng..

[56] Couture O., Hingot V., Heiles B., Muleki-Seya P., Tanter M. (2018). Ultrasound Localization Microscopy and Super-Resolution: A State of the Art. IEEE Trans. Ultrason. Ferroelect. Freq. Contr..

[57] Christensen-Jeffries K., Brown J., Aljabar P., Tang M., Dunsby C., Eckersley R.J. (2017). 3-D In Vitro Acoustic Super-Resolution and Super-Resolved Velocity Mapping Using Microbubbles. IEEE Trans. Ultrason. Ferroelectr. Freq. Control.

[58] Hansen L., Rath A., Amin Naji M., McDermott A., Sørensen C.M., Kjer H.M., Gundlach C., Dahl A.B., Jensen J.A., Mehrmohammadi M., Boehm C. (2025). Assessment of Super-Resolution Ultrasound Imaging Using the Erythrocytes through Comparison with Micro-CT. Proceedings of the Medical Imaging 2025: Ultrasonic Imaging and Tomography.

[59] Cohen J.F., Korevaar D.A., Altman D.G., Bruns D.E., Gatsonis C.A., Hooft L., Irwig L., Levine D., Reitsma J.B., de Vet H.C.W. (2016). STARD 2015 Guidelines for Reporting Diagnostic Accuracy Studies: Explanation and Elaboration. BMJ Open.

